# Supplementary material for: Tracking cortical representations of facial attractiveness using time-resolved representational similarity analysis
Source: Sci Rep. 2020 Oct 8;10:16852. doi: 10.1038/s41598-020-74009-9 (PMC7546608; doi:10.1038/s41598-020-74009-9)
Supplement: Supplementary file 1 — Supplementary Information. [file 41598_2020_74009_MOESM1_ESM.pdf]

Supplementary Information

Tracking cortical representations of facial attractiveness using time-resolved representational similarity analysis

Daniel Kaiser, Karen Nyga

Supplementary Contents Page

Figure S1. Further analysis of information shared between attractiveness judgments and neural representations 2

Figure S2. Correspondence between DNN features and brain representations 3

Figure S3. N170 event-related potential results 4

Figure S4. Summary of face metadata 5

Figure S5. Comparison of individual participants' attractiveness judgements and database ratings 6

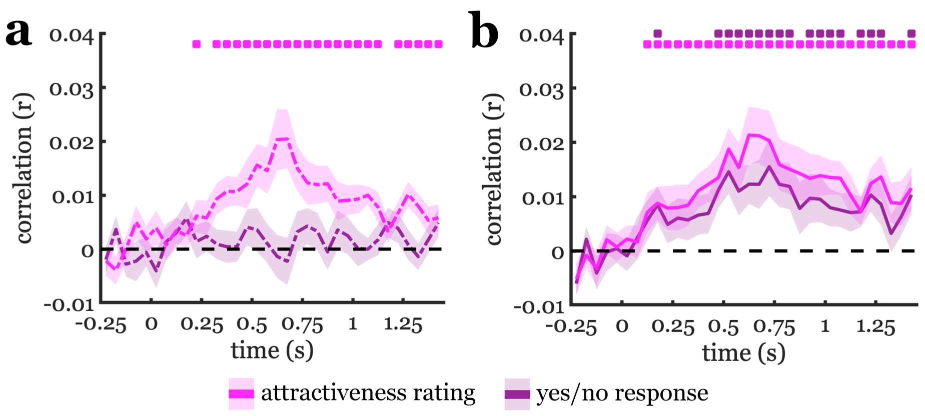

Figure S1. Further analysis of information shared between attractiveness judgments and neural representations. a) Comparing the information shared between neural representations and the yes/no responses and attractiveness ratings. When partialing out individual participants' yes/no responses, their individual attractiveness ratings still significantly predicted neural representations, suggesting that the attractiveness ratings add fine-grained information beyond the yes/no responses. By contrast, partialing out the attractiveness ratings did remove the correlation between yes/no responses and the neural data. b) Controlling for average attractiveness judgments in the experiment. When partialing out the average yes/no responses and attractiveness ratings (across all participants in our experiment) from participants' individual judgements, we found that individual judgments still predicted cortical representations, from 150-200ms and from 100-150ms for the yes/no responses and attractiveness ratings, respectively. This result supports the conclusion that early representations of facial attractiveness to some degree are individually specific. Error margins represent standard errors of the mean. Significance markers denote $p<0.05$ (corrected for multiple comparisons across time).

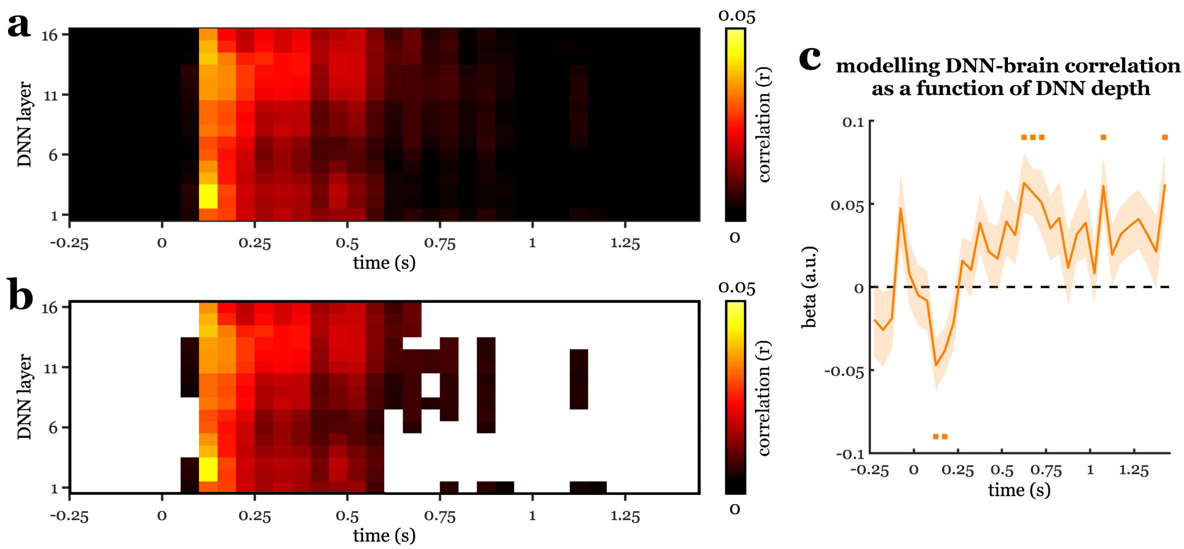

Figure S2. Correspondence between DNN features and brain representations. a) Correlation of RDMs extracted from each DNN layer and neural RDMs at each time point. b) Same as (a), with correlations thresholded at $p<0.05$ (corrected for multiple comparisons across time). c) Across processing time, different depths of the network are expected to yield the best predictions for the brain data; particularly, early DNN layers should explain early processing better, and late DNN layers should explain late processing better. To test this prediction, for each participant and time point, we predicted the correlation between the DNN model RDMs and the neural RDMs as a function of the layers' depth. We found that during early processing (100-200ms), network depth was negatively related to the DNN-brain correspondence, showing that earlier layers predicted neural representations better. This effect reversed during later processing, where the later layers predicted neural representations better. Significance markers denote $p<0.05$ (corrected for multiple comparisons across time).

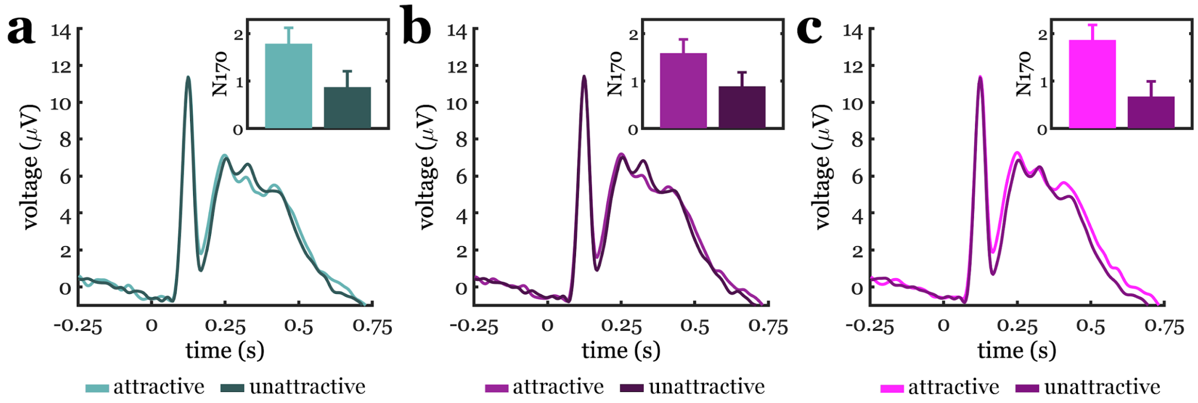

Figure S3. N170 event-related potential results. ERPs were averaged for electrodes P8/PO8 and P7/PO7. We then compared these ERPs to attractive faces and unattractive faces. This was done in three ways: (a) Comparing responses to faces whose database rating was above or below the median database rating, (b) comparing responses to faces where individual participants responded with yes or no on the attractiveness response (if responses were inconsistent across repetitions, we considered the more frequently chosen response), and (c) comparing responses to faces whose individual attractiveness rating was above or below the median of the respective participant's ratings. Inlays show peak N170 voltages (at 168ms post-stimulus) for the attractive and unattractive faces. For all three conditions, we found a significant N170 voltage difference, with a stronger N170 amplitude (i.e., lower voltage) for the less attractive faces (database ratings: t[22]=2.77, p=0.011; yes/no responses: t[22]=2.39, p=0.026; attractiveness ratings: t[22]=3.74, p=0.001). Error bars represent standard errors of the difference. Note that for the ERP analyses an additional low-pass filter at 30Hz was applied to the data.

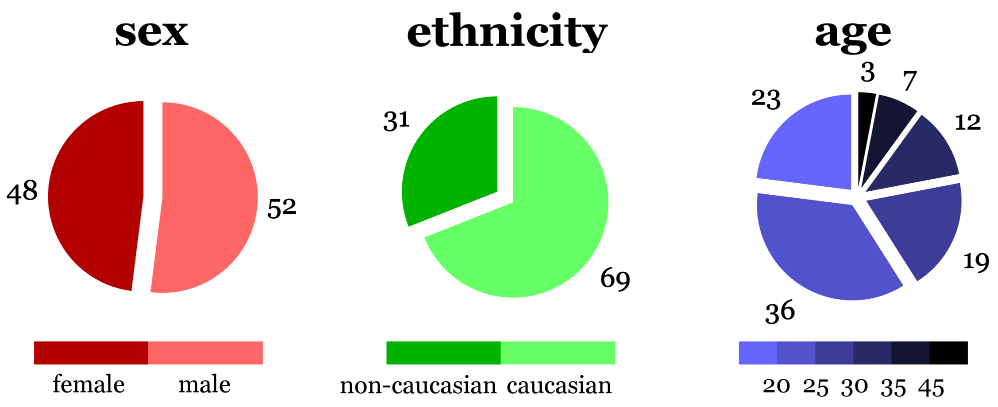

Figure S4. Summary of face metadata. Pie charts show the distribution of face sex, ethnicity and age across the 100 faces used in the experiment.

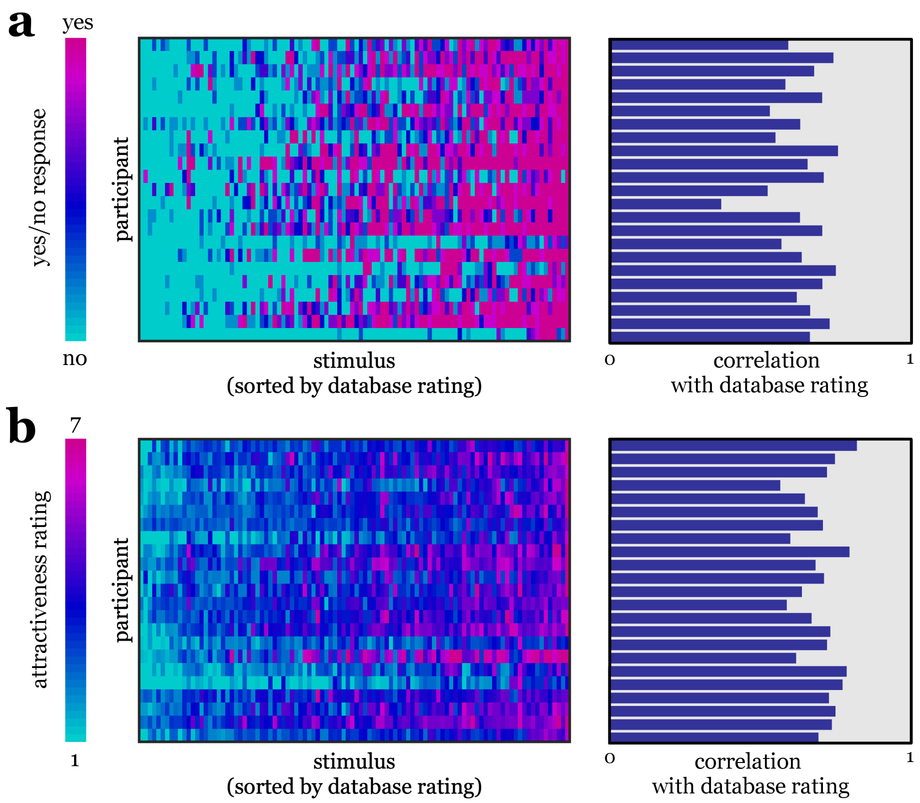

Figure S5. Comparison of individual participants' attractiveness judgements and database ratings. Colour-coded plots show individual participants' yes/no responses (a) and attractiveness ratings (b) for the 100 face stimuli used in the experiment. On the x-axis, stimuli are sorted by their average rating in the Face Research Lab London Set, from low attractiveness to high attractiveness. Bar plots show the correlation of each participants' yes/no responses (a) and attractiveness ratings (b) with the database ratings, across the 100 stimuli.
